# Crystalline Biomimetic Calcium Phosphate Coating on Mini-Pin Implants to Accelerate Osseointegration and Extend Drug Release Duration for an Orthodontic Application

**DOI:** 10.3390/nano12142439

**Published:** 2022-07-16

**Authors:** Menghong Li, Gang Wu, Mingjie Wang, Ernst B. Hunziker, Yuelian Liu

**Affiliations:** 1 Department of Oral Cell Biology, Academic Centre of Dentistry Amsterdam (ACTA), University of Amsterdam and Vrije Universiteit Amsterdam, 1081 LA Amsterdam, The Netherlands; m.li@acta.nl (M.L.); g.wu@acta.nl (G.W.); m.wang@acta.nl (M.W.); 2 Centre of Regenerative Medicine for Skeletal Tissues, Department of Clinical Research, University of Bern, 3010 Bern, Switzerland; ernst.hunziker@dbmr.unibe.ch; 3 Group for Bone Biology, Department of Clinical Research, University of Bern, 3010 Bern, Switzerland

**Keywords:** biomimetic material, calcium phosphate, bovine serum albumin, osteointegration, bone implant contact

## Abstract

Miniscrew implants (MSIs) have been widely used as temporary anchorage devices in orthodontic clinics. However, one of their major limitations is the relatively high failure rate. We hypothesize that a biomimetic calcium phosphate (BioCaP) coating layer on mini-pin implants might be able to accelerate the osseointegration, and can be a carrier for biological agents. A novel mini-pin implant to mimic the MSIs was used. BioCaP (amorphous or crystalline) coatings with or without the presence of bovine serum albumin (BSA) were applied on such implants and inserted in the metaphyseal tibia in rats. The percentage of bone to implant contact (BIC) in histomorphometric analysis was used to evaluate the osteoconductivity of such implants from six different groups (n=6 rats per group): (1) no coating no BSA group, (2) no coating BSA adsorption group, (3) amorphous BioCaP coating group, (4) amorphous BioCaP coating-incorporated BSA group, (5) crystalline BioCaP coating group, and (6) crystalline BioCaP coating-incorporated BSA group. Samples were retrieved 3 days, 1 week, 2 weeks, and 4 weeks post-surgery. The results showed that the crystalline BioCaP coating served as a drug carrier with a sustained release profile. Furthermore, the significant increase in BIC occurred at week 1 in the crystalline coating group, but at week 2 or week 4 in other groups. These findings indicate that the crystalline BioCaP coating can be a promising surface modification to facilitate early osseointegration and increase the success rate of miniscrew implants in orthodontic clinics.

## 1. Introduction

Miniscrew implants (MSIs) refer to the screws that are temporarily implanted into alveolar bone to provide absolute anchorage for orthodontic tooth movement [1]. They can provide maximal anchorage control with minimal patient compliance and are widely used to correct various orofacial deformities and malocclusions [1,2,3]. In most cases, immediate loading is usually applied on MSIs, as primary stability can be achieved right after implantation through the mechanical interlocking between MSIs and alveolar bone [4]. However, for the patients with low bone density, such as teenagers, the primary stability is usually insufficient to support the anchorage function of MSIs. Subsequently, the immediate loading is of high risk, which results in a relatively high failure rate (22.86%) [5,6,7,8,9,10]. In such cases, a delayed loading at 2 or 4 weeks is advised in orthodontic clinics to allow the gradual establishment of secondary stability. The biological basis for secondary stability is the establishment of a direct connection between MSIs and living bone, also known as osteointegration, during the bone remodeling process [6,11,12,13]. As a high-quality osteointegration is a critical factor to determine the loading occasion and to ensure the long-term success of MSIs [14], many efforts have been made to accelerate and enhance the osteointegration of MSIs with an aim to enable an early loading and reduce the failure rate [15,16,17,18,19,20,21].

One of the promising methods to promote the osteointegration of metallic implants is the biomimetic coating [22], which was originally introduced by Kokubo and his colleagues in 1990. With this original biomimetic coating method, metallic implants can be coated with a layer of apatite by being immersed into a simulated body fluid (SBF). However, the application of this original biomimetic coating technique is limited by its long immersion period (approximately 1–2 weeks) and the need for active chemical groups for the formation of the apatite layer. To overcome these limitations, a two-phase biomimetic calcium phosphate (BioCaP) coating technique was developed by Prof. Klass de Groot in 2001. The preparation process of this BioCaP coating includes the formation of an initial amorphous biomimetic calcium phosphate (BioCaP) coating after soaking metallic implants in a five-fold SBF for 24 h at 37 °C [23]. With the amorphous BioCaP as a seeding layer, the crystalline coating is formed after the metallic implants are immersed in a calcium phosphate super-saturated (CPS) solution for 48 h at 37 °C [24,25,26,27]. The amorphous property is symbolized by a broad peak at 2θ = 28°–32° with calcium-to-phosphorus ratios (Ca/P) from 1.53 to 1.64, whereas the crystalline layer bears a characteristic narrow peak close to 2θ = 26° and a broader one at 2θ = 32° [26] with the Ca/P range of 1.37–1.45. In our previous study, we showed that the volume fraction of total bone, especially unmineralized bone matrix in the crystalline BioCaP coating group, was more than that of the uncoated group at one week [28]. On the other hand, the amorphous CaP coating is also of great potential in accelerating osteoconductivity as it has been shown that the amorphous CaP coating bears a stronger capacity of stimulating in vitro intracellular mineralization—a marker for terminal osteoblastic differentiation—compared to the crystalline CaP one [29]. Hitherto, the application potential of our amorphous (and crystalline) BioCaP coating in promoting and accelerating the osteointegration of implants has not been compared.

Coating properties can be further modified by protein incorporation. In our previous study, bovine serum albumin (BSA) was biomimetically co-precipitated with Ca^2+^ and PO_4_^3−^ ions of the crystalline BioCaP coating, which led to a significant enhancement of mechanical strength [23] and resistance to the shear forces [30]. A study from another group further showed that the new bone area in dog muscles in the BSA-incorporated CaP coating group was significantly larger than that of the CaP coating alone group [31]. These studies suggest that the incorporation of BSA may be an effective method to improve the biological performance of BioCaP coatings.

In this study, we aimed to sort out the optimal BioCaP coating that may be applied to accelerate the osteointegration and facilitate the early loading of MSIs in orthodontic treatment. For this purpose, we first characterized the physicochemical properties of, and the cellular response to, the amorphous and crystalline BioCaP coating with or without incorporated BSA. Thereafter, we compared the time-course osteointegration process of titanium mini-pin implants furnished with these coatings in a model of metaphyseal tibial implantation in rats.

## 2. Materials and Methods

### 2.1. Experimental Design

#### 2.1.1. Design of the Mini-Pin Implant

**Figure 1:**
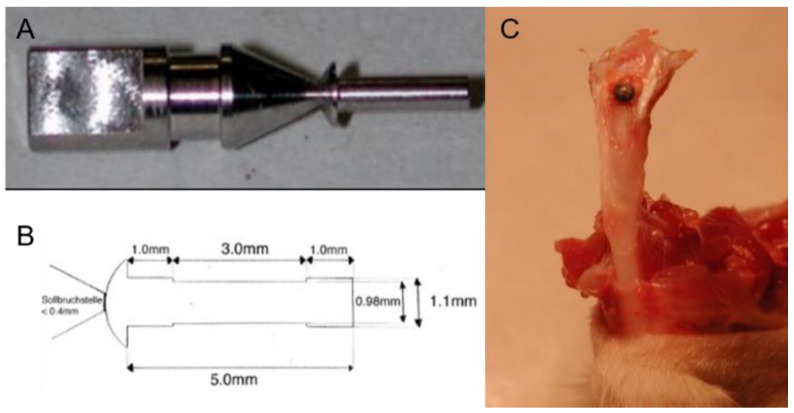
(**A**) Graph depicting the holder and cap of the mini-pin implant, (**B**) the design of the mini-pin implant, and (**C**) the implantation of the mini-pin implant.

In this study, we adopted the novel sandblasted, large-grit, acid-etched (SLA) titanium (Ti) mini-pin implant (Figure 1A,B). It was designed by W. Hofstetter at the University of Bern and locally applied into the trabecular bone of the tibial metaphysis of adult female rats (Figure 1C) to mimic clinical MSIs. The diameter of the groove in the middle (0.98 mm) was smaller than both ends (1.1 mm). Hole preparation was performed at the implantation site followed by the insertion of the mini-pin implant. After operation, the connection of the mini-pin implant and holder, which was located on the top of the cap, could be stopped. The chamber (3 mm in length) in the middle part of the mini-pin implant allowed us to observe the time-course-establishing process of bone-implant interaction This also ensured that the coating area was isolated from the surrounding tissue and subsequently provided an assessable field for bone formation after implantation.

#### 2.1.2. Coating Procedure on Titanium Pin

To prepare the amorphous coating, Ti pins were immersed in five-fold SBF (684 mM NaCl; 12.5 mM CaCl_2_·2H_2_O; 21 mM NaHCO_3_; 5 mM Na_2_HPO_4_·2H_2_O, 20 mL/pin) for 24 h at 37 °C under high nucleation conditions, viz., in the presence of 7.5 mM MgCl_2_·2H_2_O, to inhibit crystal growth. The fine, dense layer of amorphous BioCaP was thereby formed and served as a seeding surface for the deposition of a crystalline layer.

To prepare the crystalline coating, Ti pins were immersed in a modified five-fold-concentrated simulated body fluid (684 mM NaCl; 13.4 mM KCl; 9 mM CaCl_2_·2H_2_O; 60 mM NaHCO_3_; 2 mM Na_2_HPO_4_·2H_2_O, 20 mL/pin) for 24 h at 37 °C under high nucleation conditions, viz., in the presence of 5 mM MgCl_2_·2H_2_O, to inhibit crystal growth. After deposition of the first (amorphous) layer, some of the samples were dried and sterilized for future characterization. The second (crystalline) layer was produced by immersing the samples in a sterilized supersaturated solution of calcium phosphate (40 mM HCl; 2 mM Na_2_HPO_4_·2H_2_O; 4 mM CaCl_2_·2H_2_O; 50 mM Tris base; 136 mM NaCl; at pH 7.4 (20 mL/pin)).

By the groups that were to be functionalized by BSA (Sigma, Saint Louis, MO, USA, A9647), the proteins were introduced either into the five-fold SBF for the amorphous coating, or into the supersaturated calcium phosphate solution for crystalline coating at a final concentration of 0.1 mg/mL.

### 2.2. In Vitro Study

#### 2.2.1. Characterization of Coatings

Coated implants in the absence or presence of incorporated BSA were examined in a scanning electron microscope (SEM, XL 30, Philips, Eindhoven, The Netherlands). For this purpose, the materials were mounted on aluminum specimen stubs and sputtered with gold particles to a thickness of 10–15 nm.

The chemical property of the coating pattern, as well as the absence or the presence of incorporated BSA, was evaluated by Fourier-transform infrared spectroscopy (FTIR).

A confocal laser scanning microscope (CLSM) was used to reveal the distribution of amorphous and crystalline coatings, and the incorporation of BSA into the coatings. In this study, fluorescein isothiocyanate-conjugated BSA (FITC-BSA) was used to substitute for BSA (0.1 mg/mL). During the preparation of amorphous and crystalline coatings, rhodamine B (0.1 mg/mL) and FITC-BSA were simultaneously introduced into the respective coating solutions. After thorough rinsing and freeze-drying, the coated samples were embedded in methyl methacrylate. Sections that were 600 mm thick were prepared and affixed to Plexiglas holders. These sections were then ground to a thickness of 80 mm for inspection in a CLSM equipped for fluorescence imaging (Zeiss LSM 510 META CLSM with LSM 510 Acquisitions software and Image 3D software, Heidelberg, Germany). As the two fluorescent markers emitted signals at different wavelengths, we were able to track separately the distribution of the coating (red) and BSA incorporated (green).

#### 2.2.2. Loading and Release Kinetics of BSA In Vitro

Three mini-pin implants furnished with each coating in the absence and presence of FITC-BSA were incubated in sealed 10 mL glass tubes containing 2 mL of 1 M phosphate-buffered saline (PBS, pH 7.4) at 37 °C under agitated conditions (60 times/min). Aliquots of the medium (containing released protein) were withdrawn at specific time intervals up to 35 days (n = 3). The other 3 mini-pin implants of each coating type were immersed in 1 mL of 0.5% EDTA (pH 8.0) and vortexed twice for 5 min. These samples were used to determine the total protein content per sample. Fluorescence was measured in a spectrophotometer (excitation wavelength: 485 nm; emission wavelength: 519 nm). Fluorescence readings were converted to amounts of protein using a standard curve, which was generated by preparing a dilution series of FITC-BSA in 5 mL of PBS.

#### 2.2.3. Alkaline Phosphatase (ALP) Activity of Primary Osteoblasts

Primary osteoblasts were isolated from rat calvaria by sequential collagenase digestion. Primary osteoblasts were grown until 90% confluency in α-MEM (Minimum Essential Medium) supplemented with 10% Fetal Bovine Serum (FBS) and 1% Pen/Strep. The culture medium was changed every three days. Cells were seeded on coated Ti discs. After culturing for 8 days, the discs were washed by phosphate-buffered saline (PBS) 3 times and the activity of ALP in cells was measured by p-nitrophenyl phosphate substrate reactions. In brief, after culturing for the indicated periods, cells were washed twice with PBS and incubated in 50 µL of 0.2% Triton X-100 37 °C for 40 min. Then, the cells were incubated with 100 µL of substrate (10 mmol/L of p-NPP and 1 mmol/L of MgCl_2_) for 30 min at 37 °C. The reaction was stopped by adding 100 µL of 1 mol/L of NaOH. The p-nitrophenol formed was spectrophotometrically measured at 405 nm using a microplate reader.

### 2.3. In Vivo Study

#### 2.3.1. Experiment Grouping

Six experimental groups (including the control group) were established, with n = 6 rats per group. The samples were retrieved at 3 days, 1 week, 2 weeks, and 4 weeks post-surgery. Hence, the total number of rats utilized was 144 (6 groups × 6 rats per group × 4 time-points).

Group 1: No coating no BSA (naked implants).

Group 2: No coating Ads. BSA. Implants bearing passively adsorbed BSA (overnight in PBS containing 0.1 mg/mL of BSA).

Group 3: Amorphous coating. Implants bearing an amorphous coating.

Group 4: Amorphous coating Inc. BSA. Implants bearing an amorphous coating containing 0.1 mg/mL of BSA.

Group 5: Crystalline coating. Implants bearing a crystalline coating.

Group 6: Crystalline coating Inc. BSA. Implants bearing a crystalline coating containing 0.1 mg/mL of BSA.

#### 2.3.2. Surgical Procedure

All animal experiments followed the guidelines of the central animal facility of the medical faculty in Bern. This study was approved by the University of Bern, Switzerland with number BE18/08 and all animal experiments complied with the Directive 2010/63/EU. A total of 144 female Wistar rats with an average weight of 200–220 g and average age of 8 months were housed under customary conditions at a controlled temperature (20 °C) and a light/dark cycle (12/12 h).

All of the rats were separately anesthetized through retroperitoneal injections of ketamine (20 mg/kg) and xylazine (2 mg/kg) and were given a mixture of 20% *v/v* isoflurane followed by the propylene glycol for inhalation. The surgical site was shaved first and rubbed with iodine, and the muscles were separated over the tibia to expose the periosteum.

An exact opening was made at the tibia bone by making a hole (0.9 mm in diameter and 5 mm in length) using the drilling machine. The drilling must be performed with adequate irrigation with saline solution to minimize the temperature rise in the bone. Then, the implant was inserted with a “press-fit” into the hole and the skin was sutured well. All of the animals were given penicillin with a dose of 400,000 units as an antibiotic for three postoperative days. The animals were euthanized at the experimental time points with CO_2_. The tibia was then harvested and the position of the implant located was recorded by a radiograph (Faxitron).

#### 2.3.3. Histological Process

Mini-pin implants and the immediately surrounding tissue were excised and fixed in 10% formaldehyde for several days. Following rinsing and dehydration in alcohol, the specimens were embedded in methyl methacrylate. Using a systematic random sampling protocol, the embedded specimens were cut into slices using a diamond saw. These saw-cuts were mounted on plexiglass holders, polished, and surface-stained with basic Fuchsine, Toluidine blue O, and McNeil’s Tetrachrome.

#### 2.3.4. Histomorphometric Analysis

A well-established two-step systematic sampling was applied to all the samples at a certain final magnification (′380). Around 20 pictures per sample were obtained in a Nikon-Eclipse light microscope and printed in color. The BIC was analyzed on these colored prints using the intersection-counting methodologies.

### 2.4. Statistical Analysis

All data were presented as mean ± standard deviations. Statistical analyses were carried out with GraphPad Prism 5.0 (GraphPad Software, San Diego, CA, USA). Comparisons between groups were performed by one-way analysis of variance (ANOVA). Tukey’s post hoc multiple comparisons analysis was performed to determine any significant differences between groups. A value of *p* < 0.05 was considered statistically significant.

## 3. Results

### 3.1. Coating Characterization

**Figure 2:**
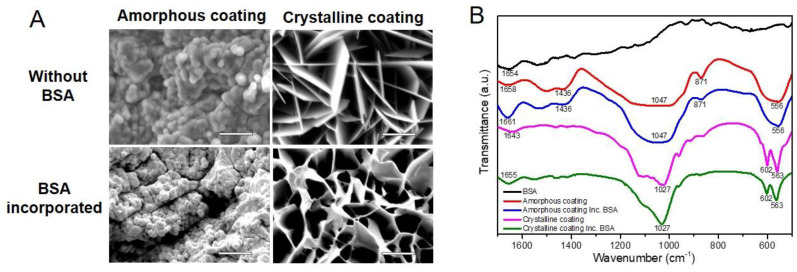
(**A**) Micrographs of scanning electron microscopy depicting the surface morphology of biomimetic calcium phosphate coatings deposited on mini-pin implants (scale bars = 5 μm). (**B**) Fourier-transform infrared spectra for amorphous and crystalline coatings in the absence or presence of incorporated bovine serum albumin (BSA).

According to micrographs of SEM (Figure 2A), the amorphous layer showed a dense, noncrystalline morphology, which was deposited in the form of spherical particles. The incorporation of BSA in the amorphous coating did not significantly change the morphology of the amorphous coating. The crystalline layer showed a rhomboid plate-like crystalline morphology and the incorporation of BSA changed the rhomboid plates into curly plates. These SEM micrographs were consistent with our previous ones characterized by XRD analysis [30].

FTIR was carried out to re-characterize the chemical structures of coatings with or without the incorporation of BSA. As shown in Figure 2B, the amorphous coatings were characterized by two bands that appeared at 1047 cm^−1^ and 556 cm^−1^, corresponding to PO_4_^3−^; one at 871 cm^−1^, corresponding to HPO_4_^2−^; one at 1436 cm^−1^, corresponding to CO_3_^2−^ [32,33]. Based on these data, the amorphous seeding layer can be classified as carbonated calcium phosphate. The incorporation of BSA in the amorphous coating did not cause any significant distinction in the above-mentioned bands. Crystalline coatings were characterized by a twin band at 602 cm^−1^ and 563 cm^−1^, which corresponds to O-P-O bending, and a single one at 1027 cm^−1^, which corresponds to P-O stretching [34,35]. These bands and stretching modes of the P-O group reflect the crystalline nature of this calcium phosphate layer [36] and were not changed by the incorporation of BSA. However, there were two bands undergoing a shift to higher wavenumbers after being coprecipitated with BSA [37]. One was at 1658 cm^−1^ in the amorphous coating, and the other was at 1643 cm^−1^ in the crystalline coating, representing molecular water of the calcium phosphate layer [38]. In addition, in the spectrum of BSA itself, a band was apparent at this position (1654 cm^−1^) and, in the case of the protein, corresponds to C=O stretching in amide-I groups (-CO-NH2). The shift to a higher-wavelength position that occurred in the spectra of coatings represents the incorporation of BSA. The structures were reconfirmed and showed consistence with our previous results [26].

**Figure 3:**
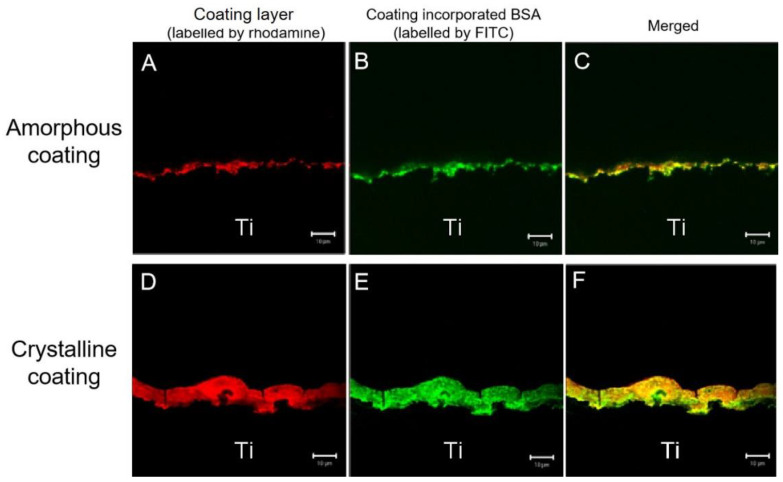
Confocal laser-scanning images of amorphous coating layer on the surface of titanium discs labeled with rhodamine (**A**), the location of fluorescein isothiocyanate conjugated-bovine serum albumin (FITC-BSA) (**B**), and the distribution of FITC-BSA in the coatings (merged images) (**C**) and crystalline coating layer (**D**–**F**). The two sets of images were merged in the third horizontal panel (scale bars = 10 μm).

In the CLSM, the rhodamine-labeled amorphous and crystalline layers were revealed to follow the surface contours of the cross-sectioned mini-pin implants (red signal in Figure 3A,D). Figure 3A,B are from the same sample. Figure 3D,E are also from the same sample. It showed that the crystalline layer was approximately 7 times thicker than the amorphous one. The green fluorescence signal recorded the positions of FITC-BSA (in Figure 3B,E). The merger of the two images (red and green signals) revealed that FITC-BSA was homogeneously distributed throughout both amorphous and crystalline layers (yellow signal in Figure 3C,F). These images showed that FITC-BSA was colocalized with coatings layers.

### 3.2. Loading and Release Kinetics of BSA In Vitro

**Figure 4:**
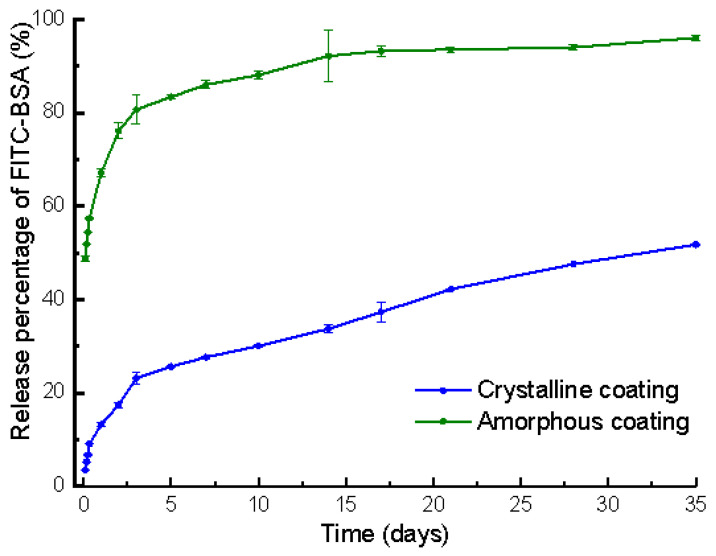
Graph depicting the temporal release profiles of fluorescein isothiocyanate conjugated-bovine serum albumin (FITC-BSA) incorporated in amorphous and crystalline coatings on mini-pin implants. Mean values (n = 3 for each coating) were presented together with the standard deviation.

The total loading of FITC-BSA in the amorphous and crystalline coating of each mini-pin implant was 8.37 ± 0.16 μg and 88.15 ± 2.15 μg (mean ± SD, n = 3), respectively. The release behaviors of the FITC-BSA deposited on the amorphous and crystalline coatings were evaluated for up to 35 days. It comprised an initial burst-release phase spanning 3 days and a subsequent gradual release at a steady rate from the 3rd day to the 35th day. During the initial 3 days, 81% and 23% of the FITC-BSA loaded was released from the amorphous and crystalline coatings, at a rate of 27% and 7% per day, respectively. By the end of the monitoring period, 96% and 52% of the FITC-BSA loaded was depleted from amorphous and crystalline coatings, respectively (Figure 4). During the 3rd day to 35th day, the proportion of BSA released from the crystalline coating was 29%, almost two times more than that of the amorphous coating (15%).

### 3.3. In Vitro Cellular Experiments

**Figure 5:**
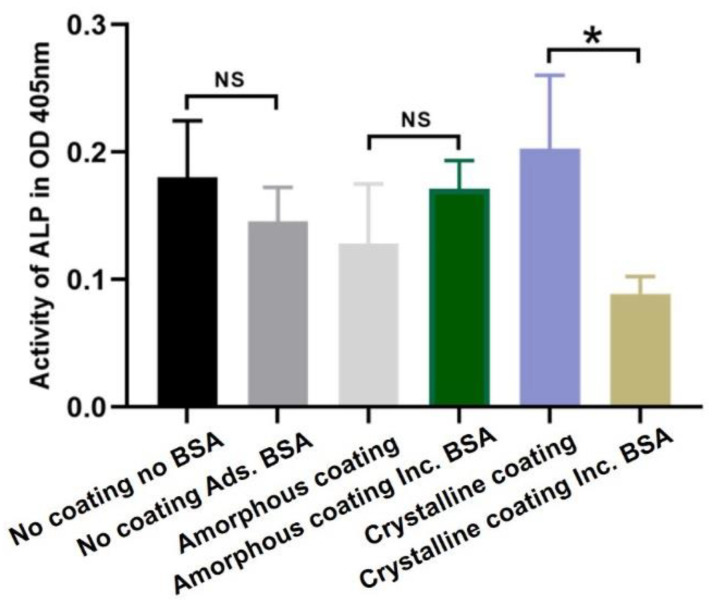
Graph depicting relative alkaline phosphatase (ALP) activity in primary osteoblasts after 8 days of culture in different groups. Mean values (n = 3 for each group) were presented together with the standard deviation (* *p* < 0.05, NS *p* > 0.05).

The results of alkaline phosphatase (ALP) activity of primary osteoblasts are depicted in Figure 5. After an 8-day cell culture, no significant differences were observed in the uncoated groups and amorphous coating groups, indicating that the presence of BSA did not significantly change ALP activity in the uncoated group and the amorphous coating group. Compared with the crystalline coating alone group, the incorporation of BSA significantly decreased the ALP activity of primary osteoblasts at 8 days (*p* < 0.05).

### 3.4. In Vivo Study

**Figure 6:**
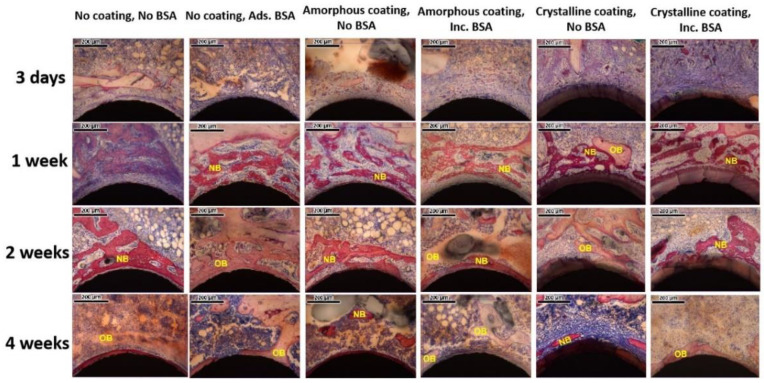
Representative light micrographs of sections from each group 3 days, 1 week, 2 weeks, and 4 weeks after implantation. NB: newly formed bone; OB: old bone (scale bars = 200 μm).

Newly formed bone and old bone were clearly visualized from representative microscopic images of histological sections at high magnification (Figure 6). At day 3, a blue cord-like osteoid was found in the two no coating groups. A similar osteoid was also detected with a lower amount in the amorphous coating groups. In the groups of the crystalline coating either with or without BSA, a thick layer (68.85 ± 1.72 μm and 69.42 ± 1.44 μm) of a light-pink-stained coating was detected on the surfaces of Ti mini-pins. Surrounding the coating, a newly formed osteoid exhibited a deep-blue color and with a morphology of branched dendrite. At week 1, in the group of no coating no BSA, the blue osteoid was deeper than that of day 3. In other groups, reddishly stained newly formed bone was detected and connected with old bone, stained in pink. They gradually formed an integrative network surrounding the mini-pin implants. In the crystalline alone group, a continuous layer of new bone formed and covered most of coating surfaces. In contrast, a thin intervening layer of connective tissue was detected between the bone layer and the coating in the group of crystalline coating with BSA. At week 2, reddishly stained newly formed bone appeared in the no coating no BSA group. In other groups, the reddish porous new bone partially changed into ossification patterns with lighter color, representing the maturation of new bone tissue. At week 4, most of the bone tissues surrounding implants were stained in pink and integrated with each other as a result of bone remodeling.

**Figure 7:**
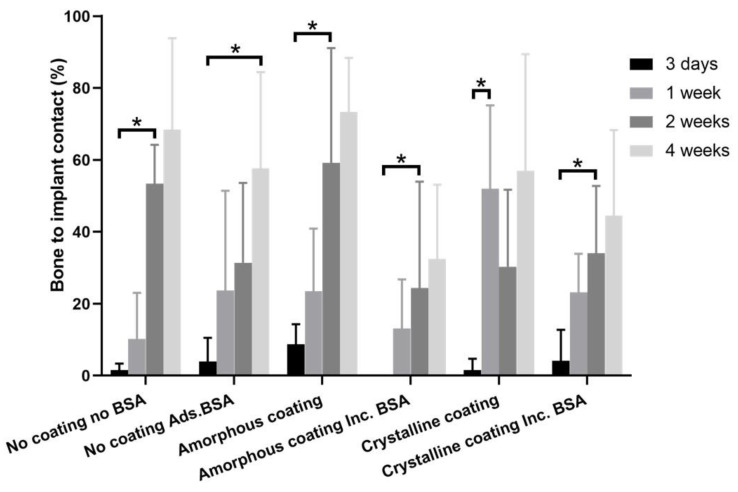
Graph depicting histomorphometric analysis of bone to implant contacts (BICs) of mini-pin implants from different groups, which were implanted in the rat tibia for 3 days, 1 week, 2 weeks, and 4 weeks. Mean values (n = 6 for each group) were presented together with the standard deviation (* *p* < 0.05).

Histomorphometric analysis (Figure 7) showed the percentage of bone to implant contact (BIC%) of different groups 3 days, 1 week, 2 weeks, and 4 weeks post-surgery.

At day 3, the BIC% in all groups still remained at a very low level, ranging from 0% to 8.70%. The BIC% at this timepoint was regarded as a baseline in each group. A significant increase in BIC% could be found as early as one week in the crystalline group (52.00 ± 23.15%) compared with that of day 3 (1.56 ± 3.13%). In comparison with the corresponding baselines, the significant increases in BIC% were found in the no coating no BSA group (53.36 ± 10.86%), amorphous coating groups (24.37 ± 29.61 and 59.19 ± 31.92%), and BSA-incorporated crystalline coating group (34.02 ± 18.72%) only at 2 weeks. In the no coating BSA adsorption group, such a significant increase in BIC% (57.68 ± 26.77%) could only be observed at week 4.

## 4. Discussion

Previous studies have shown that various biomimetic coatings are effective in promoting bone regeneration, while their efficacies in accelerating the osteointegration of MSIs remains largely unknown [27,39]. In this study, our results showed that a significant increase in BIC% could be observed as early as one week in vivo in the crystalline coating group. In contrast, a significant increase in BIC% appeared at two weeks in amorphous coating groups. Furthermore, the presence of BSA in the BioCaP coatings decreased the percentage of BIC or delayed the significant increase in BIC% (Figure S1). These findings suggest the promising application potential of the crystalline BioCaP coating in accelerating the osteointegration of MSIs in orthodontic treatment. The acceleration of osteoconduction and of osseointegration was described previously by us using coatings [28].

As the improvement of the surface properties of MSIs could promote cell proliferation and accelerate osseointegration [40,41], various surface treatments have been applied to modify both the surface composition as well as its topography to increase the success rate of MSIs in clinics [19,20,42,43,44]. One of the most common applications is the sandblasted, large-grit, acid-etched (SLA) technique [45,46], for instance, Orthosystem (Straumann, Andover, MA, USA) and C-implant (CImplant Co., Seoul, South Korea) [46,47,48,49,50]. In a split-mouth controlled trial on human subjects, it showed that the removal torque of the SLA Ti group was statistically higher than that of the control Ti group [51], accompanied with a higher survival rate. In this study, the BioCaP coatings were applied on the SLA Ti mini-pin implants and the SLA Ti mini-pin surface was regarded as the control group when comparing the acceleration effect in osteointegration.

BSA has been widely used in the biomedical field due to some advantages such as biodegradability, no (or low) immunogenicity, high drug-binding capacity, and osteogenic potential [52,53,54,55]. In our previous study, it was demonstrated that the crystallinaty of the crystalline BioCaP coating could be deformed when the concentration of BSA in the coating solution was higher than 0.1 mg/mL [56], because BSA in solution can operate as a template for oriented crystal nucleation, and act as a specific inhibitor of crystal growth [57,58,59,60]. Therefore, the concentration of BSA at 0.1 mg/mL in the coating solution was adopted in this study.

BIC% in histomorphometric analysis directly reflects osseoconduction on the bone–implant interface [61]. It has been regarded as a predictor for implant stability and survival [62,63,64]. In this study, the increase in BIC% generally showed a time-dependent manner. In the crystalline coating group, a significant increase in BIC% appeared as early as one week (52.00 ± 23.15%), which has already reached the requirement of a clinically successful implant [65]. This phenomenon suggested that the crystalline coating could significantly accelerate the osteointegration of osseous implants, thereby facilitating an earlier mechanical loading. Such an accelerating effect may be attributed to the following three mechanisms: (1) The polar functional groups, such as the hydroxyl ions and phosphate groups in the crystalline coating, make it more hydrophilic than the uncoated SLA Ti surface [26,66]. This hydrophilic surface can support the interaction between cells and the surface, benefit cell adhesion and spreading, and mediate more natural biological cell responses/behaviors [67,68,69,70,71]. (2) The crystalline coating consists of large crystals oriented vertically to the substrate surface. This structure exhibits a network structure open to the surrounding body fluid [72]. (3) As calcium phosphate is the main inorganic component of human bones, the released ions from either the amorphous or crystalline coating increase the local inorganic ion concentration and consequently enhance new bone formation adjacent to the implant surface [73,74,75,76,77,78]. As the crystalline coating is much thicker than the amorphous coating, it is supposed to provide more calcium and phosphate to continuously sustain bone formation. At week 2, the BIC% insignificantly decreased to 30.33 ± 21.41%. This could be explained by the activation of osteoclasts and the release of osteolytic proteins [79]. In addition, the degradation of the crystalline coating may also lead to the decrease in BIC% [72]. However, the BIC% reached 57.00 ± 32.42% at week 4, indicating that the insignificant decrease at week 2 did not compromise the overall osteoconduction and the process of bone remodeling reached an equilibrium state of BIC%, remaining at 50% [62,79,80].

One limitation of this study was that we did not measure the mechanical stability of the implants. Further experiments, such as the removal torques and resonance frequency analysis, should be performed to elucidate the beneficial effects of the crystalline coating on the mechanical stability and survival rate of MSIs. To further confirm this application, a larger sample size should be performed on large animals.

## 5. Conclusions

The results showed that the protein loading capacity of the crystalline BioCaP coating in each mini-pin implant was 88.15 ± 2.15 μg, ten times more than that of amorphous ones (8.37 ± 0.16 μg). Moreover, the BIC significantly increased as early as 1 week in the crystalline BioCaP coating group, whereas the increase in BIC was observed at 2 weeks or 4 weeks in other (No coating no BSA, No coating Ads. BSA, amorphous coating, amorphous coating Inc. BSA, and crystalline coating Inc. BSA) groups. These findings suggest that the crystalline BioCaP coating is a promising technique to accelerate osseointegration and increase the success rate of MSIs during orthodontic treatment.

## Data Availability

The data and contributions presented in the study are included in the article. Further inquiries can be directed to the corresponding author.

## Supplementary Materials

The following supporting information can be downloaded at: https://www.mdpi.com/article/10.3390/nano12142439/s1, Figure S1: Graph depicting comparison of bone to implant contacts (BICs) of mini-pin implants from amorphous groups. Mean values (n = 6 for each group) are presented together with the standard deviation (* *p* < 0.05).
