# Supplementary material for: Crystalline Biomimetic Calcium Phosphate Coating on Mini-Pin Implants to Accelerate Osseointegration and Extend Drug Release Duration for an Orthodontic Application
Source: Nanomaterials (Basel). 2022 Jul 16;12(14):2439. doi: 10.3390/nano12142439 (PMC9324071; doi:10.3390/nano12142439)
Supplement: Supplementary file 1 [file nanomaterials-12-02439-s001.zip › nanomaterials-1774784-supplementary.pdf]

Crystalline Biomimetic Calcium Phosphate Coating on Mini-pin Implants to Accelerate Osseointegration and Extend Drug Release Duration for an Orthodontic Application

Menghong Li[1], Gang Wu[1], Mingjie Wang[1], Ernst. B. Hunziker[2,3]， Yuelian Liu[1,*]

[1]Department of Oral Cell Biology, Academic Centre of Dentistry Amsterdam (ACTA), VU University and University of Amsterdam, Amsterdam, the Netherlands.

[2]Centre of Regenerative Medicine for Skeletal Tissues, Department of Clinical Research, University of Bern, Bern, Switzerland

[3]Group for Bone Biology, Department of Clinical Research, University of Bern, Bern, Switzerland.

***Corresponding author.** Email: y.liu@acta.nl

Supplementary Materials

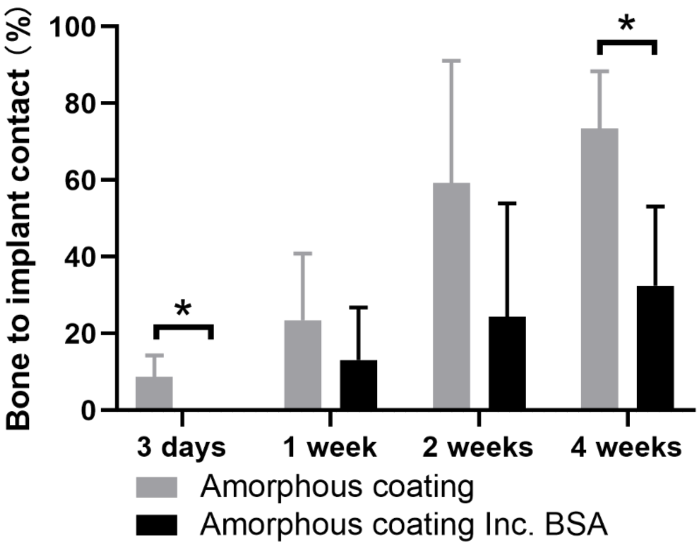

Figure S1. Graph depicting comparison of bone to implant contacts (BIC) of mini-pin implants from amorphous groups. Mean values (n=6 for each group) are presented together with the standard deviation (*P＜0.05).
